# Unanticipated Intraoperative Seizure During Craniotomy: A Case Report and Anesthetic Considerations

**DOI:** 10.7759/cureus.97638

**Published:** 2025-11-24

**Authors:** William S Jones, Ashley Tasz, Jeremy Hensley, Arpan Kohli

**Affiliations:** 1 Department of Anesthesiology, West Virginia University School of Medicine, Morgantown, USA; 2 School of Medicine, West Virginia University School of Medicine, Morgantown, USA

**Keywords:** anesthetic management, cerebral venous sinus thrombosis (cvst), craniotomy, intraoperative seizure, meningioma

## Abstract

Intraoperative seizures during cranial surgeries under general anesthesia are uncommon but clinically significant occurrences that require rapid identification and treatment. The rarity of such occurrences is underscored by the limited number of similar case reports in the literature. This report describes an incident of intraoperative seizure during craniotomy for tumor resection under general anesthesia. This case involved a lesion outside the cortical regions most often associated with seizure activity, such as the hippocampus and neocortex. We will discuss the diagnosis and management, as well as the broader incidence, diagnosis, and management of intraoperative seizures under general anesthesia.

## Introduction

Intraoperative seizures are rare but potentially life-threatening complications that require rapid recognition and management to prevent adverse outcomes. The reported incidence of seizures varies by surgical population, occurring in approximately 0.8% of all surgical cases and up to 1.8% of intracranial procedures [1,2]. However, these rates primarily reflect data from postoperative studies; intraoperative seizure events are far less frequently documented [1].

Most contemporary craniotomies are performed under total intravenous anesthesia (TIVA) using propofol, which possesses anticonvulsant properties. Nonetheless, seizures may occur secondary to cortical stimulation, electrolyte abnormalities, venous congestion, or intracranial pressure (ICP) elevations. Continuous electroencephalography (EEG) monitoring is standard practice for early detection of subclinical seizure activity, but clinical recognition may be limited when neuromuscular blockade is used [3,4].

This case describes an intraoperative seizure in a patient undergoing resection of a retroclival mass, later confirmed as a meningioma. Unlike more seizure-prone regions such as the hippocampus or neocortex, the lesion in this case was in a region not typically associated with seizure activity [5]. The patient also had no prior history of seizures and was not on antiepileptic medications, aligning with guidelines that discourage routine prophylactic use in brain tumor patients [2]. The absence of standardized protocols for intraoperative seizure treatment underscores the critical role of anesthesiologists in navigating these challenges through real-time clinical decision-making and interdisciplinary collaboration [6].

This report highlights the successful use of propofol, sevoflurane, and intraoperative cold saline to terminate the seizure, offering insights into effective management strategies and the importance of anesthesiologists’ adaptability in addressing rare intraoperative events.

## Case presentation

**Figure 1:**
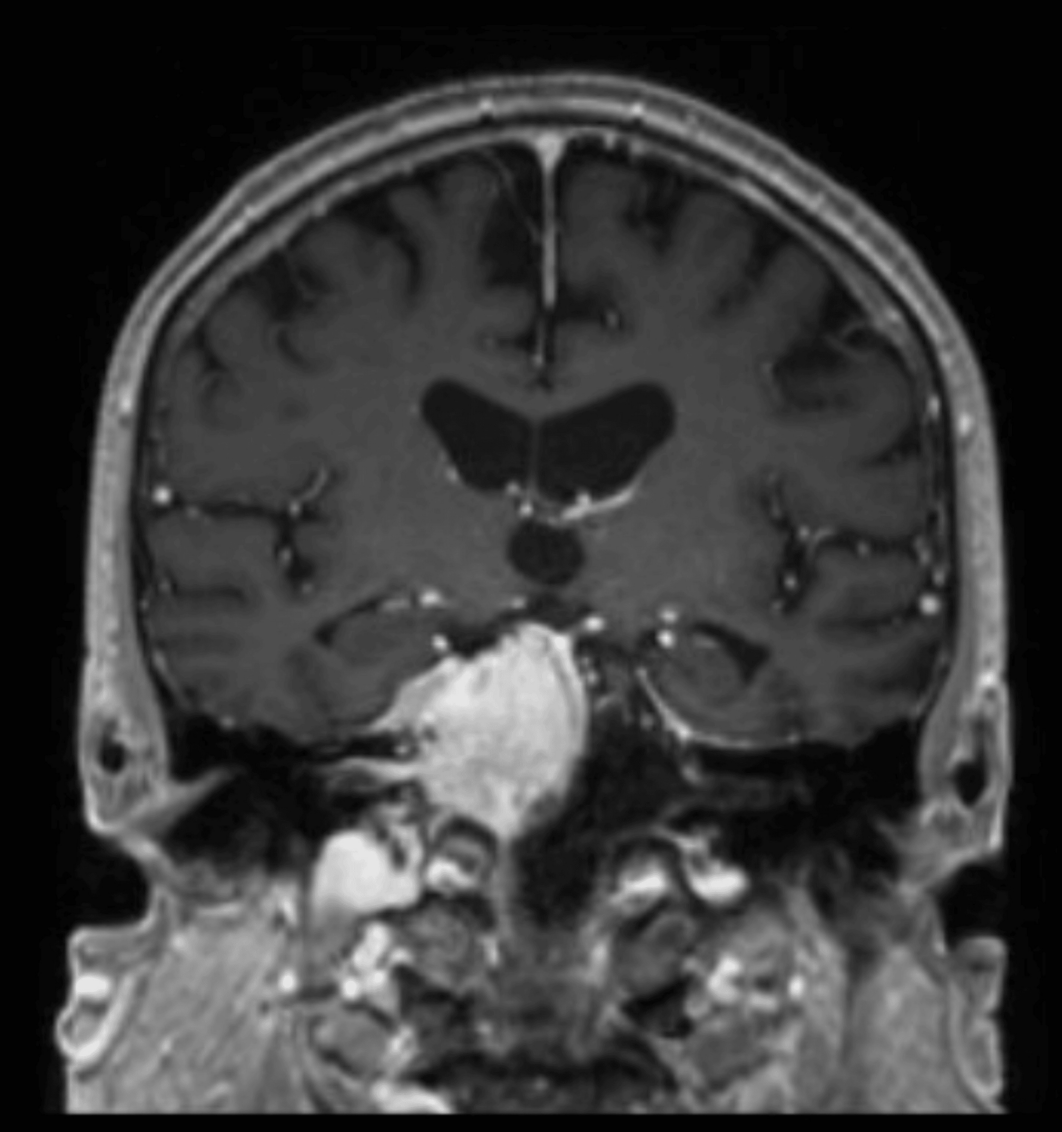
Pre-operative MRI with and without IV contrast Extra-axial mostly homogeneously enhancing mass extending into the sella and prepontine cistern with mass effect on the pons. Mass = 4.6 cm × 4.9 cm

Our patient is a 73-year-old male who was referred by an ophthalmologist after presenting with right esotropia. A subsequent brain MRI revealed a lesion anterior to the brainstem (Figure 1), with a differential diagnosis including meningioma, schwannoma, metastasis, or other neoplasms, leading to further evaluation at an outside facility. The patient was referred for surgical evaluation due to the potential for mass effect, including cranial nerve VI palsy with an inwardly directed right eye, but no reported vision changes, headaches, or neurological deficits. The patient had no history of seizures or a family history of anesthesia complications. His past medical history includes hypertension, anxiety, cataracts, alcohol use, and a history of smoking. During a follow-up neurosurgery visit, the patient reported decreased vision in the left eye, but no other new symptoms.

The patient first underwent a diagnostic cerebral angiogram. No embolic agent was administered, as arterial feeders were not safely accessible. The procedure was performed under monitored anesthesia care (MAC) with a propofol infusion. 

**Figure 2:**
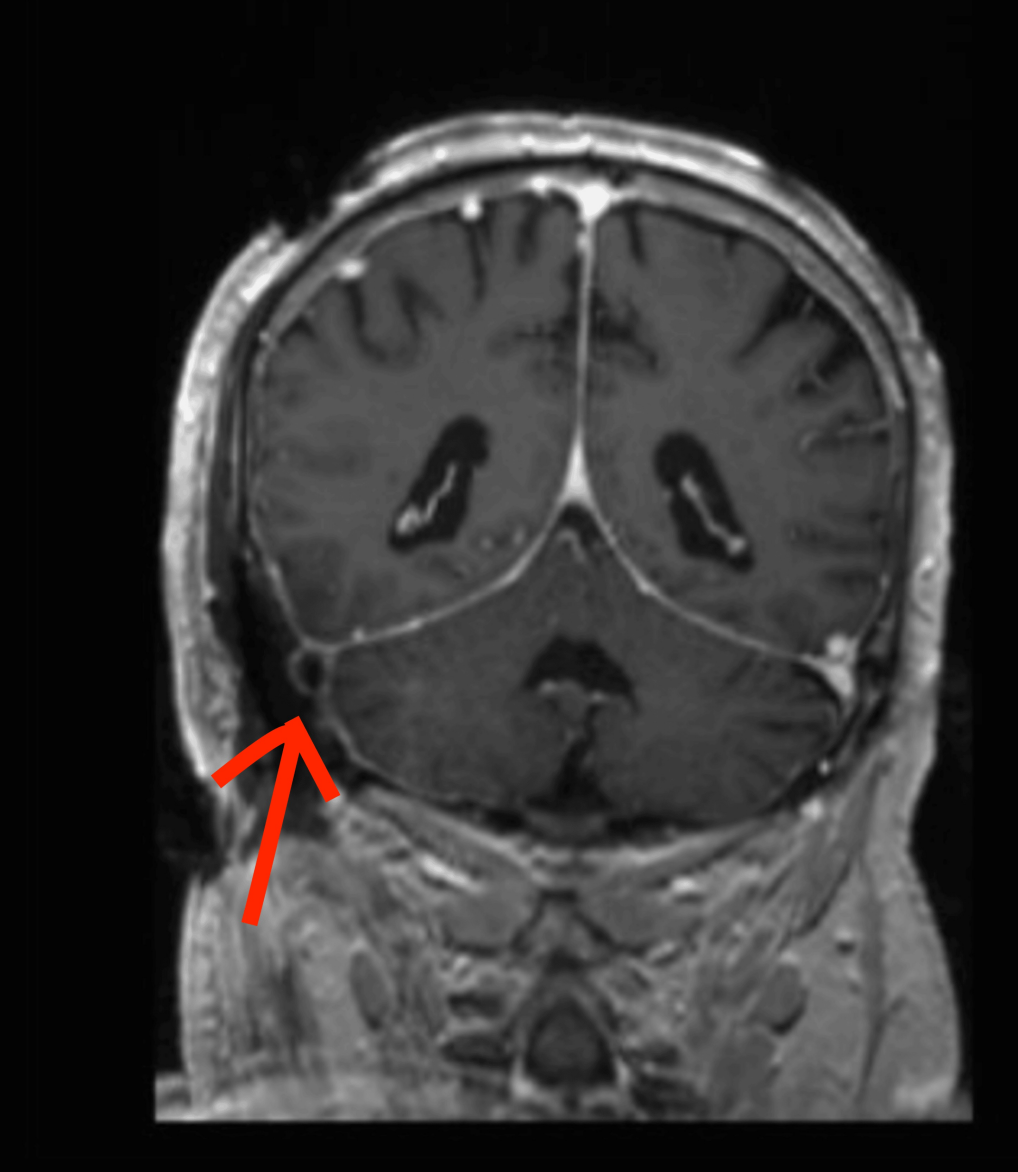
Post-operative MRI with and without IV contrast Acute thrombosis of the right transverse/sigmoid sinus seen on post-op MRI.

After attempted embolization, the patient was brought to the operating room the following day for tumor resection. Anesthesia was induced with fentanyl, lidocaine, propofol, and rocuronium and maintained with sevoflurane (<0.5 MAC), propofol, and remifentanil. A subtemporal transtentorial approach was used, and during tumor dissection, generalized convulsions were noted along with hypotension. Epileptogenic EEG activity was confirmed by the neuromonitoring technician. The episode lasted approximately 90 seconds and was initially managed with a propofol bolus (100 mg), an increased inspired concentration of sevoflurane, an increase in propofol infusion, and administration of cold saline over the dura adjacent to the operative site by the surgical team. Additional intraoperative management included the administration of a loading dose of levetiracetam (1 g). The patient remained intubated and sedated and transferred to the neurocritical care unit. Postoperatively, an MRI (Figure 2) showed acute thrombosis of the right transverse sinus, sigmoid sinus, and peripheral jugular vein, showing insight into the possible cause of the seizure. Continuous EEG for 24 hours postoperatively revealed no further epileptogenic activity. The patient was initiated on therapeutic anticoagulation with enoxaparin and continued on levetiracetam to prevent further seizures. After a stable recovery, the patient was discharged with instructions to return for further resection, as 30% of the tumor remained.

Prior to the second surgery, the patient remained seizure-free on outpatient levetiracetam and continued anticoagulation with enoxaparin. For the second procedure, a combined presigmoid transpetrosal approach was utilized. The anesthesia regimen included preoperative midazolam, induction with propofol, lidocaine, and succinylcholine, maintenance with sevoflurane (<0.5 MAC), and continuous infusions of propofol and remifentanil. Additionally, the patient received levetiracetam prior to resection. Pathology of the resected mass confirmed a WHO grade 1 meningioma.

## Discussion

Intraoperative seizures are rare events under general anesthesia, with a higher incidence in intracranial procedures. TIVA with propofol typically provides seizure suppression, but seizures may still occur from cortical stimulation, increased ICP, or vascular events such as venous sinus thrombosis [1,7]. One unique characteristic of this case is the region of the brain that was operated on, as it is not typically associated with a high incidence of seizure-like activity. The brain areas most prone to seizures are the neocortical regions and the hippocampus [8], but the tumor in this patient was not located in these regions. Another unique feature is that the patient had no prior history of seizures and was not receiving any antiepileptic medications prior to surgery. Current guidelines, as noted by the Quality Standards Subcommittee of the American Academy of Neurology, recommend against the routine prophylactic use of antiepileptic drugs in patients with brain tumors. While not used in this case, a practice survey showed that more than 70% of neurosurgeons routinely use prophylactic antiepileptics [5].

Although rare, continuous EEG monitoring remains the gold standard for intraoperative seizure detection [3]. However, overt manifestations may not be visible, underscoring the importance of vigilant anesthetic and neuromonitoring collaboration. While our patient was intubated, limiting risk for aspiration, the principal concern involved potential hemodynamic instability and increased ICP.

The anesthesia team can assist with diagnosing intraoperative seizures by collaborating with the neuromonitoring team and maintaining clear communication with the surgery team when abnormal movements are observed from the patient, as was the case here. After suspecting an intraoperative seizure, the next crucial steps include continued communication between the anesthesiologist and the surgical team.

With no current published guidelines for the treatment of intraoperative seizures, the anesthesia team in this case was prepared and successfully treated the seizure without further complications [7,9]. Several options for treating intraoperative seizures include propofol bolus (0.75-1.25 mg/kg), benzodiazepines (diazepam 0.1 mg/kg or lorazepam 0.1 mg/kg), or deepening the volatile anesthetics. Cold saline administered to the suspected origin of the seizure can also be effective [3]. After recognizing the seizure in this case, a propofol bolus and deepening of sevoflurane were administered, resulting in the cessation of seizure activity on the EEG and the resolution of convulsions. Cold saline was also administered by the neurosurgical team over the dura. Once the seizure had resolved, the patient was loaded with levetiracetam and continued to receive it in the postoperative period. The patient remained seizure-free during the postoperative course. The incidence of postoperative seizures is lower in patients with no preoperative history of seizures [7].

As discussed earlier, the postoperative MRI showed venous sinus thrombosis, which has been shown to be associated with seizures. According to one study, seizures occur in up to one-third of patients with diagnosed cerebral venous thrombosis [9]. This finding is likely the cause of the intraoperative seizure in this case.

The case was re-attempted with successful resection of the tumor, and no complications were encountered. Pathology confirmed the tumor was a meningioma. The patient experienced no further complications during his hospital course and was later discharged. This case demonstrates the importance of anesthesiologists' ability to detect, diagnose, and treat rare occurrences in the operating room.

## Conclusions

This case highlights that intraoperative seizures under general anesthesia, though rare, are clinically significant events. They may occur even in patients without prior seizure history and when lesions are located outside typical epileptogenic regions. The rarity of these occurrences is highlighted by the small number of similar case reports in the literature. This case emphasizes the critical importance of employing diverse diagnostic and monitoring strategies for intraoperative seizures, being prepared for pharmacologic intervention, and fostering clear communication between the anesthesiology and surgical teams to promptly address abnormalities and ensure patient safety.
